# Efficacy and Safety of a Newly Developed Botulinum Toxin A (MBA-P01) in Patients with Moderate-to-Severe Glabellar Lines: A Randomized, Double-Blind, Active-Controlled, Multi-Center, Phase III Study with a Subgroup Analysis on Patients with COVID-19

**DOI:** 10.3390/toxins17040160

**Published:** 2025-03-23

**Authors:** Hye Sung Han, Won-Serk Kim, Yangwon Lee, Chong-Hyun Won, Wooshun Lee, Sun Young Choi, Beom Joon Kim

**Affiliations:** 1 Department of Dermatology, Chung-Ang University Gwangmyeong Hospital, Gwangmyeong-si 14353, Republic of Korea; caudrhhs@gmail.com (H.S.H.); sun02ya@naver.com (S.Y.C.); 2 Department of Dermatology, Chung-Ang University College of Medicine, Seoul 06974, Republic of Korea; 3 Department of Dermatology, Kangbuk Samsung Hospital, Sungkyunkwan University School of Medicine, Seoul 03181, Republic of Korea; susini@naver.com; 4 Department of Dermatology, Konkuk University School of Medicine, Seoul 05030, Republic of Korea; 20050078@kuh.ac.kr; 5 Department of Dermatology, Asan Medical Center, University of Ulsan College of Medicine, Seoul 05505, Republic of Korea; drwon@amc.seoul.kr; 6 Medytox Inc., Seoul 06175, Republic of Korea; drlee@medytox.com; 7 Department of Dermatology, Chung-Ang University Hospital, Chung-Ang University College of Medicine, Seoul 06974, Republic of Korea

**Keywords:** glabellar lines, botulinum toxin A, MBA-P01, coronavirus disease

## Abstract

MBA-P01 is a newly developed botulinum toxin A (BoNT-A) product designed to provide similar clinical effects as OnabotulinumtoxinA (ONA-BoNT-A), thereby providing an alternative treatment option for glabellar lines. It is another holotoxin preparation containing BoNT-A1. This randomized, double-blind, active-controlled, multi-center, Phase III clinical trial aimed to evaluate the efficacy and safety of MBA-P01 compared with OnabotulinumtoxinA (ONA-BoNT-A). In total, 318 participants were enrolled and received 20 units of MBA-P01 or ONA-BoNT-A on the forehead and glabella. At the 4-week assessment, the primary endpoint revealed no significant difference in the improvement rate of glabellar wrinkles between the two groups, confirming the non-inferiority of MBA-P01 to ONA-BoNT-A. Furthermore, some evaluation variables showed higher improvement rates for MBA-P01 than for ONA-BoNT-A. Adverse reactions and other safety analysis results were considered acceptable. Interestingly, a subgroup analysis of patients with the coronavirus disease (COVID-19) showed that the duration of BoNT-A treatment was shorter among those who contracted COVID-19 after BoNT-A treatment compared with those who have not. The limitations of this study include the predominance of female participants and the exclusive enrollment of Korean patients. MBA-P01 is expected to be clinically useful in terms of the efficient and safe reduction of glabellar wrinkles, which will provide patients with additional treatment options.

## 1. Introduction

Looking young is an instinctive desire. With the global aging population on the rise, there is an ever-growing demand for methods to prevent facial aging. The development of facial wrinkles significantly contributes to facial aging, and these facial lines also directly affect a person’s self-confidence and social interactions [1,2,3].

Botulinum toxin type A (BoNT-A) is a neurotoxin that inhibits the activity of the underlying musculature by reducing acetylcholine release from motor nerve terminals. It has been widely used as a preventive or therapeutic option for wrinkles [4]. Currently, various brands of BoNT-A including OnabotulinumtoxinA (ONA-BoNT-A), AbobotulinumtoxinA, and IncobotulinumtoxinA are available on the market [5,6,7]. Despite the established efficacy of these products, there remains a need for alternative formulations that provide similar clinical effects while expanding accessibility and treatment options for both patients and physicians. MBA-P01 is a newly developed BoNT-A product designed to provide similar clinical effects as ONA-BoNT-A, thereby expanding treatment options for patients. It is another holotoxin preparation containing BoNT-A1. The significance of developing new BoNT-A formulations lies not in creating entirely new molecular structures but in increasing accessibility, providing physicians and patients with alternative choices that maintain efficacy and safety. Previously published data provided a comparative overview of MBA-P01 and other commercially available BoNT-A formulations, highlighting their compositional similarities, which can be referenced in the long-term extension study [8,9].

This clinical trial was a phase 3 study designed to evaluate the efficacy and safety of MBA-P01 in comparison with those of a commercial product (ONA-BoNT-A) in reducing glabellar lines.

## 2. Results

### 2.1. Patient Distribution and Characteristics (Tables S1 and S2)

A total of 318 participants were randomly assigned to the study group (159 to the MBA-P01 group [test group] and 159 to the ONA-BoNT-A group [control group]). A total of 317 patients were included in the FAS. Of these, 315 were included in PPS (Figure S1).

There were no differences in demographic information or baseline characteristics, including previous BoNT-A experience, duration from the last injection, or line severity at baseline.

### 2.2. Primary Efficacy Outcome

**Figure 1:**
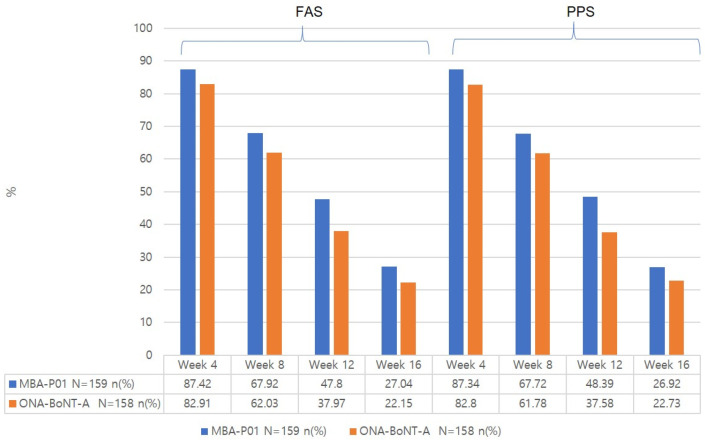
Response rate (%) at frowning by the investigator’s live assessment.

(FAS analysis) The glabellar line response rates were 87.42% (139/159) in the test group and 82.91% (131/158) in the control group (Figure 1), with no significant difference between the two groups (*p* = 0.2586). The response rate difference was 4.29% (two-sided 95% confidence interval: [−2.96 to 11.53]), and the lower limit of the one-sided 97.5% confidence interval (−2.96%) exceeded the −15% threshold (Table 1).

(PPS analysis) Similarly, the glabellar line response rate was 87.34% (138/158) in the test group and 82.80% (130/157) in the control group, with no significant difference (*p* = 0.2582). The response rate difference was 4.32% (two-sided 90% confidence interval: [−2.97 to 11.60]).

### 2.3. Secondary Efficacy Outcomes

#### 2.3.1. Response Rate at 8, 12, and 16 Weeks from the Baseline Based on the Investigator’s Live Assessment at Frowning and Rest (Table 2, Figure 1 and Figure 2)

There was no significant difference in the response rate at frowning between the two groups at any time point. The response rate decreased over time in both groups, particularly at 12 and 16 weeks after treatment.

The response rate at rest was significantly different between the groups at 16 weeks. In the FAS analysis, 16 weeks after treatment, the test group showed a 55.17% improvement, whereas the control group showed a 70.93% improvement.

**Figure 2:**
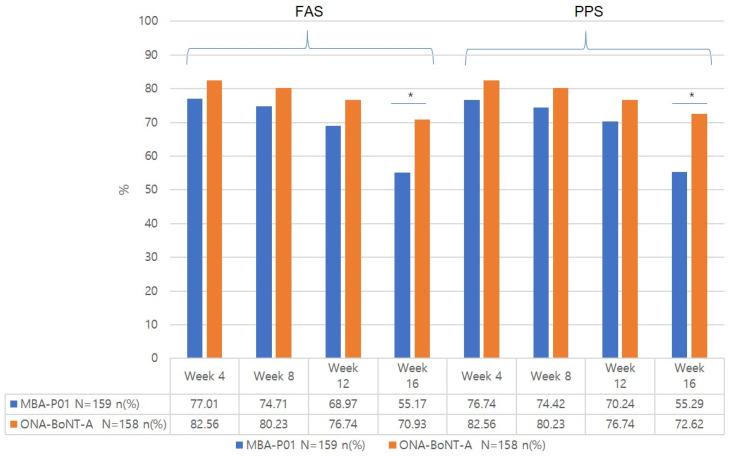
Response rate (%) at rest by the investigator’s live assessment. * *p*-value of <0.05.

**Table 2:** Illustration of glabellar line response rate (%) at frowning and at rest according to the investigator’s live assessment at weeks 4, 8, 12, and 16.

	Full Analysis Set			Per Protocol Set		
	MBA-P01 N = 159 *n* (%)	ONA-BoNT-A N = 158 *n* (%)	*p*-Value ^2^	MBA-P01 N = 158 *n* (%)	ONA-BoNT-A N = 157 *n* (%)	*p*-Value ^2^
Response ^1^ rate at frowning						
Week 4	139 (87.42%)	131 (82.91%))	0.2586 ^2^	138 (87.34%)	130 (82.80%)	0.2582 ^2^
Week 8	108(67.92%)	98(62.03%)	0.2709 ^2^	107(67.72%)	97(61.78%)	0.2700 ^2^
Week 12	76(47.80%)	60(37.97%)	0.0772 ^2^	75(48.39%)	59(37.58%)	0.0538 ^2^
Week 16	43(27.04%)	35(22.15%)	0.3120 ^2^	42(26.92%)	35(22.73%)	0.3926 ^2^
Response ^1^ rate at rest						
Week 4	67(77.01%)	71(82.56%)	0.3639 ^2^	66(76.74%)	71(82.56%)	0.3436 ^2^
Week 8	65(74.71%)	69(80.23%)	0.3850 ^2^	64(74.42%)	69(80.23%)	0.3626 ^2^
Week 12	60(68.97%)	66(76.74%)	0.2501 ^2^	59(70.24%)	66(76.74%)	0.3364 ^2^
Week 16	48(55.17%)	61(70.93%)	0.0318 ^2^	47(55.29%)	61(72.62%)	0.0190 ^2^

N = number of participants, number of participants for each item (%); ^1^ Response = FWS of glabellar lines was 0 or 1; ^2^ *p*-value between groups using Pearson’s chi-square test or Fisher’s exact test.

#### 2.3.2. Response Rate at 4, 8, 12, and 16 Weeks from the Baseline Based on the Patients’ Improvement Assessment at Frowning and Rest (Table S3, Figure 3 and Figure 4)

At 4, 8, and 12 weeks from the baseline, the response rates at frowning were significantly higher in the test group compared to the control group. However, at 16 weeks, there was no significant difference between the two groups, with response rates of 18.12% in the test group and 10.90% in the control group.

There was no significant difference in the response rate at rest between the two groups at any time point.

**Figure 3:**
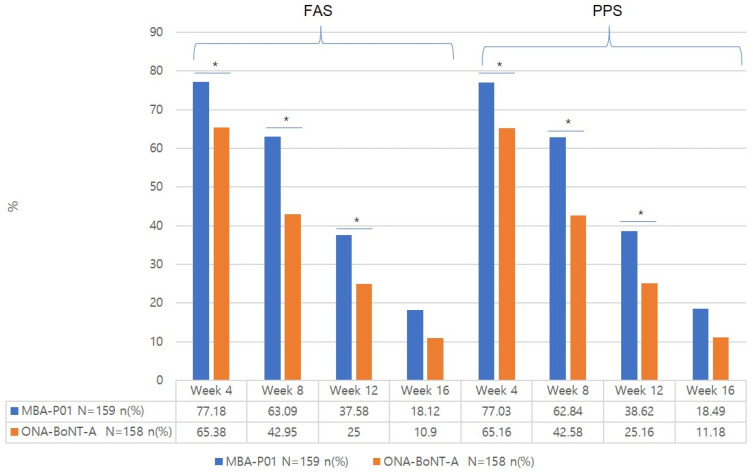
Response rate (%) at frowning by the patients’ improvement assessment. * *p*-value of <0.05.

**Figure 4:**
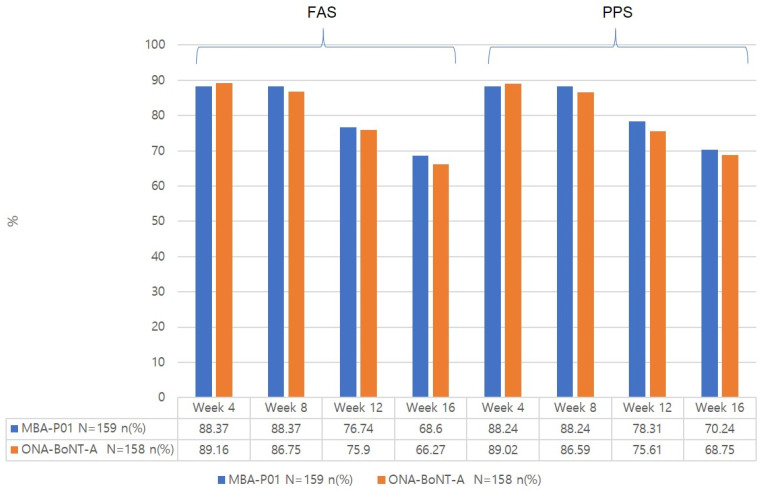
Response rate (%) at rest by the patients’ improvement assessment.

#### 2.3.3. Response Rate at 4, 8, 12, and 16 Weeks from the Baseline Based on the Independent Evaluator’s Photographic Assessment at Frowning and Rest (Figure S2)

The response rates for frowning in the test group were significantly higher than those in the control group at 8 and 12 weeks from the baseline. There was no significant intergroup difference in the response rate at 4 and 16 weeks from the baseline.

The FAS analysis showed no significant difference in the response rate at rest between the groups at any time point.

#### 2.3.4. Patients’ Satisfaction Rate at 4, 8, 12, and 16 Weeks from the Baseline Based on the Satisfaction Grade (Figure 5)

In the FAS analysis, the patients’ satisfaction rates in the test vs. control groups were 98.11% vs. 95.57% at 4 weeks, 94.97% vs. 91.14% at 8 weeks, 93.59% vs. 81.01% at 12 weeks, and 85.35% vs. 76.13% at 16 weeks. The satisfaction rate was not significantly different between the two groups at 4 and 8 weeks but was significantly higher in the test group than in the control group at 12 and 16 weeks. Throughout the entire follow-up period, more than half of the patients in both groups evaluated their satisfaction levels as “satisfied” or “very satisfied”.

**Figure 5:**
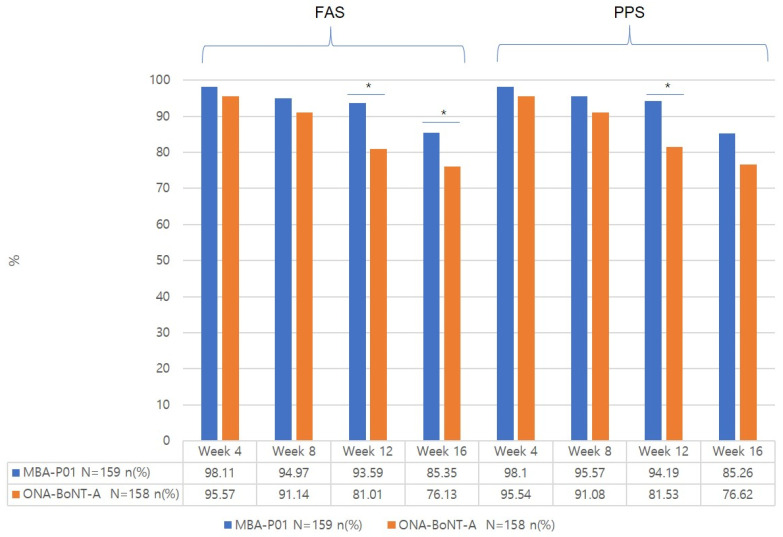
Patients’ satisfaction rates. * *p*-value of <0.05.

### 2.4. Safety Outcomes (Supplemental Tables S4–S6)

AEs occurred in 18.87% (30/159, 45 cases) of patients in the test group and 16.98% (27/159, 39 cases) of those in the control group. All AEs were TEAEs. AEs of special interest (AESIs) occurred only in the test group, with an incidence of 1.26% (2/159, 2 cases). ADRs occurred in 2.52% (4/159, 4 cases) of the test group and 0.63% (1/159, 1 case) of the control group. SAEs occurred only in the test group (0.63%, 1/159, 1 case), and there were no serious ADRs in both groups. There were no significant differences in the incidence rates of AEs, TEAEs, AESIs, ADRs, or SAEs between the two groups.

The most common TEAEs in the control group were “COVID-19” (6.92%, 11/159, 11 cases) and “headache”, “pyrexia”, and “red blood cells urine positive” (1.89%, 3/159, 3 cases). ADRs included “eyelid sensory disorder” (1.26%, 2/159, 2 cases), “injection site reaction” (0.63%, 1/159, 1 case), and “headache” (0.4%, 1/249, 2 cases) in the test group, and “injection site reaction” (0.63%, 1/159, 1 case) in the control group (Table S5).

Most TEAEs with a recorded severity were mild. None of the moderate or severe TEAEs were related to treatment (Table S6). No other significant findings were observed in the clinical or laboratory test results after administration. Additionally, no remarkable abnormal findings were detected in vital signs. Antibody testing revealed that there were no patients with antibodies identified in either the test or control group.

## 3. Discussion

There is a great variety of commercially available BoNT-A preparations, which have different efficacies and safety concerns owing to their unique biological nature [10]. Among these, ONA-BoNT-A has been the most widely used, maintaining dominance in the BoNT-A market since its approval and introduction in the United States in 1989 [11]. Therefore, most currently available information on handling BoNT-A is associated with ONA-BoNT-A [12]. This study aimed to compare the clinical efficacy and safety of MBA-P01 and ONA-BoNT-A. This study is distinct from the previously published long-term safety and efficacy study of MBA-P01 (NCT05321979), which was an extension study evaluating repeated-dose, long-term use of MBA-P01. While the extension study focused on assessing the sustained efficacy and cumulative safety profile of MBA-P01 over multiple treatment cycles, the current study presents the initial Phase III randomized controlled trial results, providing short-term efficacy, safety, and non-inferiority data compared to ONA-BoNT-A in a single-treatment setting. The response rate in the test group was 87.42% and that in the control group was 82.91% at 4 weeks; the difference in the improvement rate was not significant. Thus, it may be concluded that MBA-P01 was non-inferior to ONA-BoNT-A. Furthermore, the improvement rate of MBA-P01 observed in our study was somewhat higher than or similar to the glabellar line improvement rates with the use of other BoNT-A products confirmed in previous clinical trials conducted on Asian participants, which ranged from 78.93% to 95.2% [5,6,7,13,14].

Regarding secondary efficacy outcomes (the response rates of glabellar wrinkles when frowning, according to the investigator’s live assessment), the improvement rate in the test group was slightly higher than that in the control group at all the time points, although the difference was not significant. In both groups, the response rates showed a declining trend over time.

On the other hand, the improvement rate at rest was slightly higher in the control group than in the test group. However, we think that this does not imply that MBA-P01 is inferior to ONA-BoNT-A. Glabellar wrinkles at rest are less affected by muscle movement compared with those observed during frowning; therefore, factors that may affect them must be considered. When we examined the glabellar wrinkle scores at rest at the baseline, more patients with a score of 3 (severe) were assigned to the test group, and the difference in the score distribution between the two groups was significant. Thus, we believe that this discrepancy in the baseline severity of glabellar wrinkles at rest resulted in a lower improvement rate in the test group than in the control group.

In addition, based on the results of the patients’ satisfaction, the satisfaction rate in the test group was higher than that in the control group at all the time points, and it was maintained at a high level even at 16 weeks after administration. The satisfaction rate exceeded 90% in the test group at 12 weeks after treatment, whereas the response rate assessed by the treating investigators decreased to 47.9% at 12 weeks after treatment. Typically, the duration of action of BoNT-A preparations is approximately 3–4 months, and a similar decrease in treatment efficacy 12 weeks after treatment has been observed in other clinical trials as well [15]. For aesthetic treatments, patient satisfaction is one of the most important criteria for determining clinical efficacy, which determines whether the patient would receive retreatment [16]. The patient satisfaction of 98.11% at 4 weeks in the test group is a very high rate when compared with the results of other clinical trials on BoNT-A treatment for glabellar wrinkles (reported rates of 89.74–95%) [7,16,17,18].

As the safety of an agent used in an aesthetic procedure is of utmost importance, we compared the safety of MBA-P01 with that of the most commonly used BoNT-A product, ONA-BoNT-A. There were no significant differences between the two agents in the occurrence of AEs, ADRs, AESIs, and SAEs. ADRs in the test group included “eyelid sensory disorder”, “injection site reaction”, and “headache”. These AEs have been commonly reported for other approved BoNT-A [19].

Additionally, this study includes a COVID-19 subanalysis, which was not part of the extension study. Given that the trial period coincided with the COVID-19 pandemic, we were able to analyze the impact of COVID-19 infection on BoNT-A duration and efficacy. Although there are few reports on the interaction between BoNT-A injection and COVID-19 or vaccination, sub-acute hypersensitivity to the initial toxin treatment or a shorter interval between injections following COVID-19 vaccination has been documented [20,21,22,23]. In a retrospective study assessing the influence of the COVID-19 vaccine on the efficacy of BoNT-A injections, the mean interval between BoNT-A injections before the COVID-19 vaccination was 118.64 days, whereas the same interval after vaccination was 95.95 days [23]. Interestingly, in our study, the response rate of patients who contracted COVID-19 during the clinical trial, the response rates were 93.75% at 4 weeks, 75.00% at 8 weeks, 31.25% at 12 weeks, and 0.00% at 16 weeks (Figure S3. Compared with the response rate of all patients in the clinical trial, the response rate was higher at 4 and 8 weeks in the COVID-19 group but significantly decreased from week 12, and there were no responders at week 16. Although the sample size was very small, with only 16 patients in the COVID-19 group, we found it worthwhile to mention the singularities associated with BoNT-A treatment and COVID-19. This exploratory analysis offers unique insights into the potential interactions between viral infections and BoNT-A metabolism, a topic of growing interest in aesthetic and therapeutic neuromodulation research.

The limitations of this study include the predominance of female participants and the exclusive enrollment of Korean patients. The total study duration was 16 weeks. Therefore, additional studies with larger sample sizes and extended follow-up periods are needed to enhance the generalizability of our findings.

## 4. Conclusions

In conclusion, in this study, we showed the non-inferiority of MBA-P01 compared with ONA-BoNT-A, and some evaluation variables showed higher improvement rates with MBA-P01. Furthermore, the AEs and other safety analysis results were considered acceptable. The results support the role of MBA-P01 as a safe and effective alternative to existing BoNT-A products, expanding available treatment options for patients. Notably, the COVID-19 subanalysis provides unique insights into potential alterations in BoNT-A efficacy post-infection, highlighting an area for future research. Together with long-term extension studies, these findings contribute to a comprehensive understanding of MBA-P01’s efficacy and safety in aesthetic applications.

## 5. Materials and Methods

Study design/ethics approval

This study was designed as a randomized, double-blind, active-controlled, multi-center, Phase III clinical trial conducted across four tertiary hospitals in South Korea between August 2021 and March 2022. This study was conducted in accordance with the principles of good clinical practice and regulatory requirements. This study was approved by each institutional review board at each center. This study is registered in ClincalTrials.gov (NCT05059587). The objective was to demonstrate the non-inferiority of MBA-P01 to ONA-BoNT-A in reducing moderate-to-severe glabellar lines.

Participants

The inclusion criteria used in this study were as follows: male and female patients with an age of 19–65 years and facial wrinkle scale (FWS) scores ≥ 2 (moderate) for glabellar lines at maximum glabellar frown. Key exclusion criteria included previous BoNT-A injections within six months prior to study enrollment, hypersensitivity to botulinum toxin or its components, neurological disorders affecting facial muscles, pregnancy, or breastfeeding. The exclusion criteria used in this study were identical to those reported in the long-term extension study, where they have been detailed in full [9].

Randomization and blinding

Participants were randomly assigned (1:1) to receive either MBA-P01 or ONA-BoNT-A. Randomization was performed via a computer-generated sequence, stratified by study site. Both participants and investigators remained blinded to treatment allocation throughout this study.

Treatment protocol

Each participant received a total dose of 20 units (U) of BoNT-A, injected intramuscularly across five standardized glabellar sites (four units per site). Injections were performed using a 30-gauge syringe by trained dermatologists. The methodology was consistent between both treatment groups to ensure comparability. The standardized injection protocol followed in this study was identical to that reported in the previously published long-term extension study, where further details can be referenced [9].

Efficacy assessment

The primary objective of this study was to demonstrate the non-inferiority of MPA-P01 versus ONA-BoNT-A based on the response rate at 4 weeks, as assessed by the investigator’s live assessment at frowning. The response rate was defined as an FWS score improvement ≥ 2, with an FWS score of 0 or 1 at each evaluation time point. The outcome assessment scale and definitions used in this study were identical to those detailed in the long-term extension study [9].

Secondary efficacy assessments

1. The response rates at 8, 12, and 16 weeks from the baseline were based on the investigator’s live assessment at frowning and rest.
2. The response rates at 4, 8, 12, and 16 weeks from the baseline were based on the patients’ improvement assessment at frowning and rest.
3. The response rates at 4, 8, 12, and 16 weeks from the baseline were based on an independent evaluator’s photographic assessment at frowning and rest.
4. The participants’ satisfaction rates at weeks 4, 8, 12, and 16 from the baseline were based on their satisfaction grade. Scores ≥ 5 were considered “satisfaction”.

The response rate at frowning was defined as an FWS score improvement ≥ 2, with an FWS score of 0 or 1 at each evaluation time point.

The response rate at rest was defined based on those with an FWS score of 0 or 1 at each evaluation time point.

Safety outcomes

For safety evaluation, adverse reactions (AEs), laboratory tests, vital signs, antibody formation tests, and pregnancy tests were performed after the administration of the investigational drug. All AEs were coded using the Medical Dictionary for Regulatory Activities software (version 24.0). Blood tests, vital signs, and physical examinations were also performed. BoNT-A antibody tests were performed at the final visit.

Statistical analysis and data presentation

All statistical analyses were conducted using SAS^®^ version 9.4 (SAS Institute, Cary, NC, USA), with a *p*-value of <0.05 considered statistically significant.

Efficacy was assessed using the full analysis set (FAS) and per-protocol set (PPS), while safety was evaluated using the safety set. For the primary efficacy endpoint, the difference in improvement rates (%) between the test and control groups was analyzed using a one-sided 97.5% confidence interval based on a normal approximation (Z-distribution). If the lower limit of the one-sided confidence interval was greater than −15%, it indicated that MBA-P01 was non-inferior to ONA-BoNT-A. For secondary efficacy endpoints, the test and control groups were compared using Pearson’s chi-square or Fisher’s exact tests.

For safety assessment, AEs, including treatment-emergent AEs (TEAEs), injection site adverse reactions, adverse drug reactions (ADRs), and serious AEs (SAEs), were presented as frequencies and percentages for each treatment group and compared using Fisher’s exact test.

## Figures and Tables

**Table 1:** A summary of the glabellar lines response rates (%) at frown and at week 4 from the baseline, based on the investigator’s live assessment.

Full Analysis Set	MBA-P01 N = 159 *n* (%)	ONA-BoNT-A N = 158 *n* (%)
Response ^1^	139 (87.42%)	131 (82.91%)
Treatment Difference ^2^ [two-sided 95% CI]	4.29 [−2.96, 11.53]	
Non-inferiority	Yes	
Per Protocol Set	MBA-P01 N = 158 *n* (%)	ONA-BoNT-A N = 157 *n* (%)
Response ^1^	138 (87.34%)	130 (82.80%)
Treatment Difference ^2^ [two-sided 95% CI]	4.32 [−2.97, 11.60]	
Non-inferiority	Yes	

N = number of participants, number of participants for each item (%); CI = confidence interval; ^1^ Response = FWS of glabellar lines was 0 or 1; ^2^ confidence interval for difference between MBA-P01 and ONA-BoNT-A using CMH (Cochran–Mantel–Haenzsel)-weighted Wald C.

## Data Availability

The original contributions presented in this study are included in the article/Supplementary Material. Further inquiries can be directed to the corresponding author(s).

## Supplementary Materials

The following supporting information can be downloaded at: https://www.mdpi.com/article/10.3390/toxins17040160/s1, Figure S1: Flow diagram; Figure S2: The response rate (%) at rest by an independent evaluator’s photographic assessment; Figure S3: The response rate (%) at frowning by the investigator’s live assessment in the patients with coronavirus disease; Table S1: Illustration of characteristics of enrolled patients; Table S2: Illustration of baseline glabellar line severity at maximum frowning; Table S3: Illustration of glabellar line response rate (%) at frowning and at rest according to the subjects’ improvement assessment at weeks 4, 8, 12, and 16; Table S4: Illustration of summary of adverse events; Table S5: Illustration of adverse drug reactions (ADRs) in TEAE; Table S6: Illustration of adverse events and severity summary.

## Abbreviations

The following abbreviations are used in this manuscript:

DOAJ	Directory of open access journals
LD	Linear dichroism
TLA	Three- letter acronym
