# Supplementary material for: Efficacy and Safety of a Newly Developed Botulinum Toxin A (MBA-P01) in Patients with Moderate-to-Severe Glabellar Lines: A Randomized, Double-Blind, Active-Controlled, Multi-Center, Phase III Study with a Subgroup Analysis on Patients with COVID-19
Source: Toxins (Basel). 2025 Mar 23;17(4):160. doi: 10.3390/toxins17040160 (PMC12031167; doi:10.3390/toxins17040160)
Supplement: Supplementary file 1 [file toxins-17-00160-s001.zip › toxins-3488348-supplementary.pdf]

Supplementary Materials: Efficacy and Safety of a Newly Developed Botulinum Toxin A (MBA-P01) in Patients with Moderate-to-Severe Glabellar Lines: A Randomized, Double-Blind, Active-Controlled, Multi-Center, Phase III Study with a Subgroup Analysis on Patients with COVID-19

Hye Sung Han, Won-Serk Kim, Yangwon Lee, Chong-Hyun Won, Wooshun Lee, Sun Young Choi and Beom Joon Kim

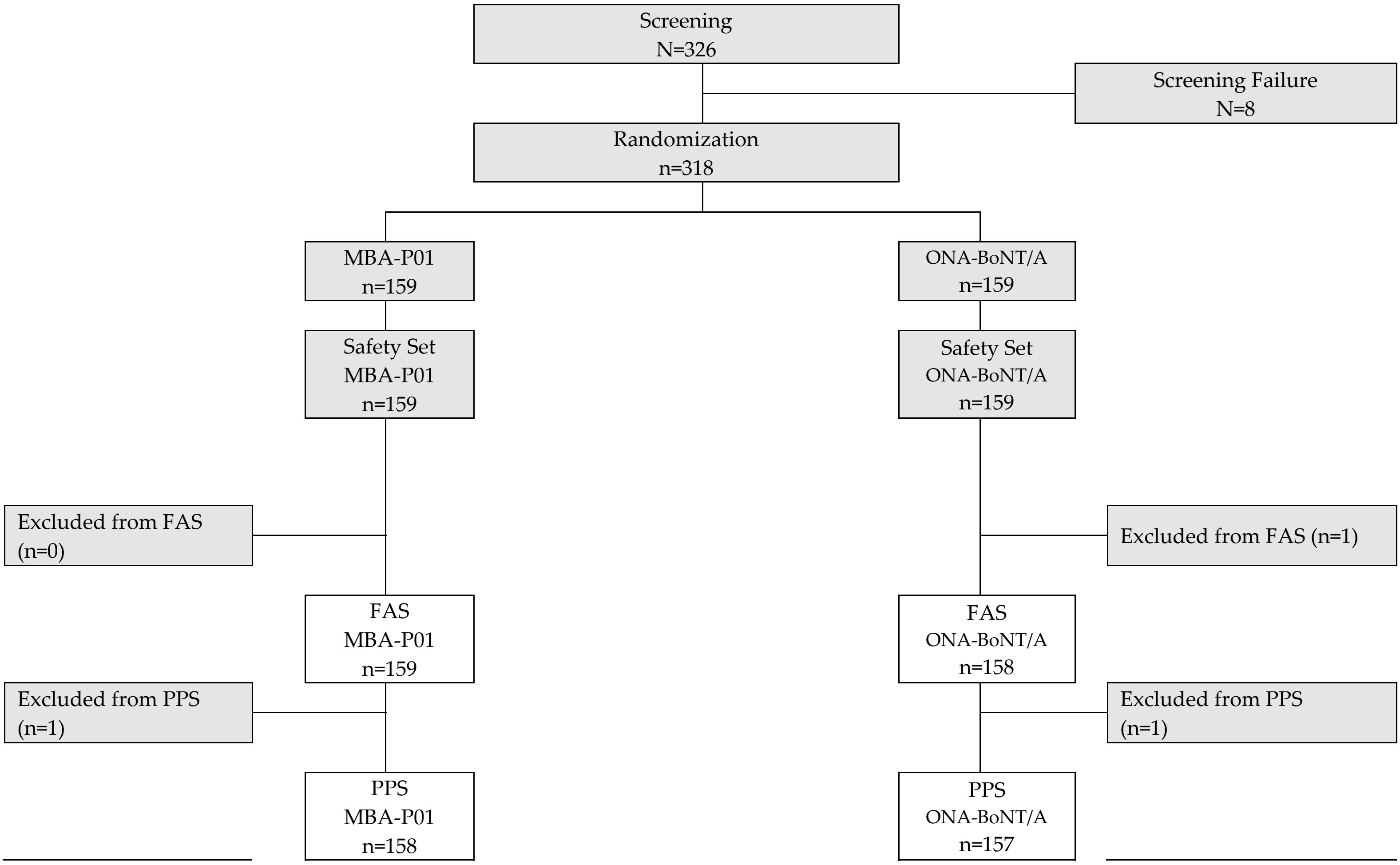

Figure S1. Flow Diagram.

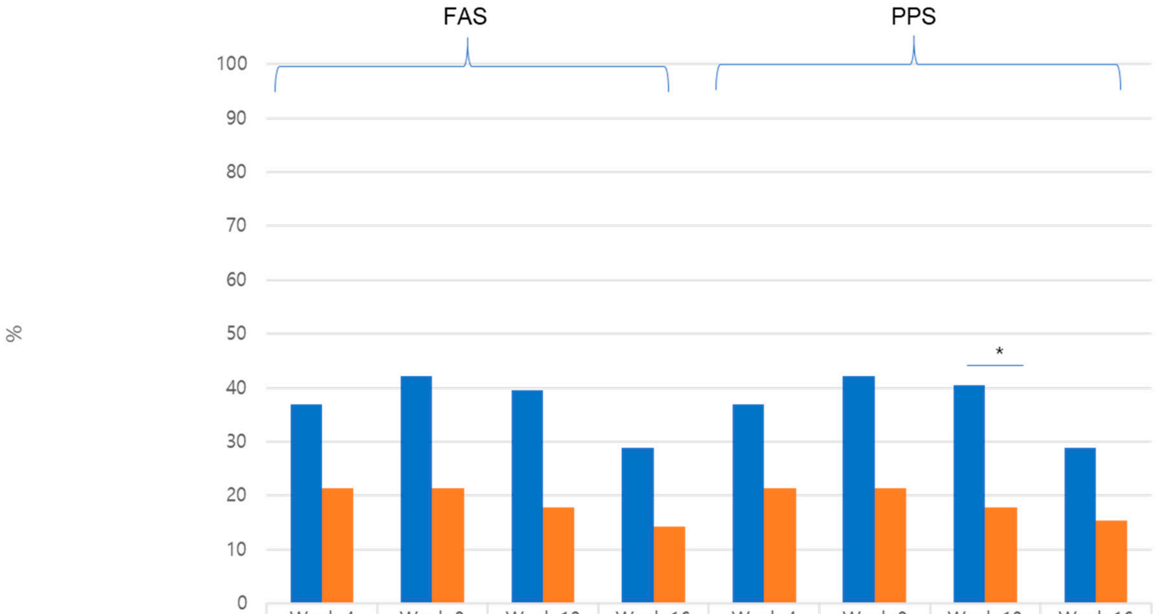

	Week 4	Week 8	Week 12	Week 16	Week 4	Week 8	Week 12	Week 16
MBA-P01 N=159 n(%)	36.84	42.11	39.47	28.95	36.84	42.11	40.54	28.95
ONA-BoNT-A N=158 n(%)	21.43	21.43	17.86	14.29	21.43	21.43	17.86	15.38

■ MBA-P01 N=159 n(%) ■ ONA-BoNT-A N=158 n(%)

Figure S2. The response rate (%) at rest by independent evaluator's photographic assessment.

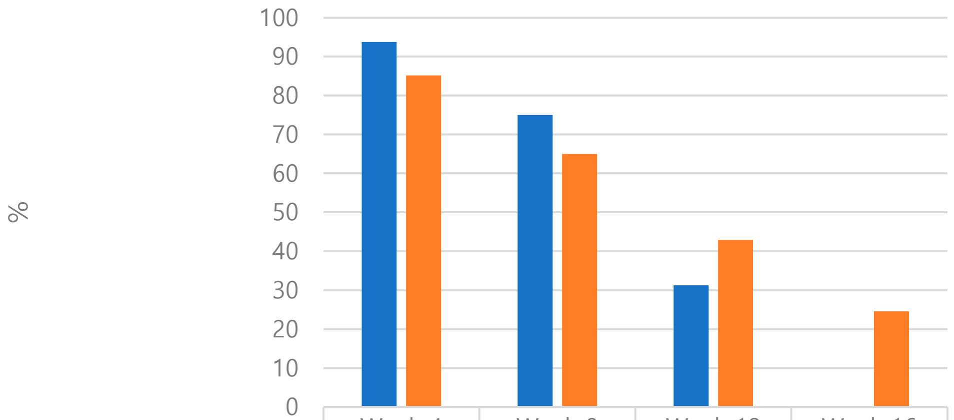

	Week 4	Week 8	Week 12	Week 16
COVID-19 infection N=16	93.75	75	31.25	0
Total subjects N=317	85.17	64.98	42.90	24.61

■ COVID-19 infection N=16 ■ Total subjects N=317

Figure S3. The response rate (%) at frowning by the investigator's live assessment in the patients with coronavirus disease.

Table S1. illustrates characteristics of enrolled patients.

		MBA-P01 N=159 n(%)	ONA-BoNT-A N=158 n(%)	Total N=317 n(%)	**p-value**
**Age (Year)**	N	159	158	317	0.3558‡
	Mean±SD	44.87±10.09	45.80±9.46	45.33±9.78	
	Median	44.00	45.00	44.00	
	Min, Max	22.00,65.00	21.00,64.00	21.00,65.00	
Age Group	19-29 years	12(7.55%)	10(6.33%)	22(6.94%)	0.6040§
	30-39 years	36(22.64%)	27(17.09%)	63(19.87%)	
	40-49 years	62(38.99%)	65(41.14%)	127(40.06%)	
	50-59 years	34(21.38%)	43(27.22%)	77(24.29%)	
	60-65 years	15(9.43%)	13(8.23%)	28(8.83%)	
Sex	Female	126(79.25%)	125(79.11%)	251(79.18%)	0.9770§
	Male	33(20.75%)	33(20.89%)	66(20.82%)	
Previous BoNT-A experience	No	93(58.49%)	101(63.92%)	194(61.20%)	0.3209§
	Yes	66(41.51%)	57(36.08%)	123(38.80%)	
Duration from the last injection (DAY)	N	66	57	123	0.3912‡
	Mean±SD	270.0±109.3	274.8±127.5	272.2±117.6	
	Median	237.0	212.0	229.0	
	Min, Max	182.0,715.0	176.0,638.0	176.0,715.0	

Abbreviations: N=Number of FAS, n: Number of subjects in the category, Min=Minumum, Max=Maximum, SD=Standard Deviation, FAS=Full Analysis Set In case of continuous variable;
p-value for ANOVA(†) or Kruskal-Wallis test(‡) up to the normality result In case of categorical variable;
p-value for Chi-square test(§) or Fisher's exact test(§§)

Table S2. illustrates baseline glabellar line severity at maximum frowning. Baseline Score at Maximum Frown (FAS).

	FWS scale	**MBA-P01 N=159 n(%)**	**ONA-BoNT-A N=158 n(%)**	**Total N=317 n(%)**	**p-value**
Investigator	2, Moderate	78(49.06%)	78(49.37%)	156(49.21%)	0.9559§
	3, Severe	81(50.94%)	80(50.63%)	161(50.79%)	
Subject	1, Mild	10(6.29%)	2(1.27%)	12(3.79%)	0.0433§
	2, Moderate	99(62.26%)	111(70.25%)	210(66.25%)	
	3, Severe	50(31.45%)	45(28.48%)	95(29.97%)	
Sex	Male	126(79.25%)	125(79.11%)	251(79.18%)	0.9770§
	Female	33(20.75%)	33(20.89%)	66(20.82%)	

Abbreviations: N=Number of FAS, n: Number of subjects in the category, FAS=Full Analysis Set
Note1: A 4-point grading FWS was used to evaluate wrinkle severity in the glabellar line;
FWS scale:0(=None);1(=Mild);2(=Moderate);3(=Severe);
§ p-value is calculated on chi-square test(or Fisher's exact test)

Table S3. illustrates glabellar line response rate (%) at frowning and at rest according to the subjects' improvement assessment at weeks 4, 8, 12, and 16.

	Full Analysis Set			Per Protocol Set		
	**MBA-P01 N=159 n(%)**	**ONA-BoNT-A N=158 n(%)**	*p*-value[2]	**MBA-P01 N=158 n(%)**	**ONA-BoNT-A N=157 n(%)**	*p*-value[2]
**Response [1] rate at frowning**						
Week 4	115(77.18%)	102(65.38%)	0.0230§	114(77.03%)	101(65.16%)	0.0229§
Week 8	94(63.09%)	67(42.95%)	0.0004§	93(62.84%)	66(42.58%)	0.0004§
Week 12	56(37.58%)	39(25.00%)	0.0177§	56(38.62%)	39(25.16%)	0.0123§
Week 16	27(18.12%)	17(10.90%)	0.0727§	27(18.49%)	17(11.18%)	0.0754§
**Response [1] rate at rest**						
Week 4	76(88.37%)	74(89.16%)	0.8718§	75(88.24%)	73(89.02%)	0.8725§
Week 8	76(88.37%)	72(86.75%)	0.7488§	75(88.24%)	71(86.59%)	0.7478§
Week 12	66(76.74%)	63(75.90%)	0.8977§	65(78.31%)	62(75.61%)	0.6800§
Week 16	59(68.60%)	55(66.27%)	0.7455§	59(70.24%)	55(68.75%)	0.8361§

N=Number of subjects, Number of subjects for each item (%)
[1] Response=FWS of glabellar lines was 0 or 1
§ p-value between groups using Pearson's chi-square test or Fisher's exact test

Table S4. illustrates summary of adverse events.

	**MBA-P01 N=159 n(%)[case]**	**ONA-BoNT-A N=159 n(%)[cases]**	**Total N=319 n(%)[cases]**	***p*-value**
Any TE	30(18.87%), [45]	27(16.98%), [39]	57(17.92%), [84]	0.6609§
Any TEAE	30(18.87%), [45]	27(16.98%), [39]	57(17.92%), [84]	0.6609§
Any AESI in TEAE	2(1.26%), [2]	0(0.00%), [0]	2(0.63%), [2]	0.4984§§
Any ADR in TEAE	4(2.52%), [4]	1(0.63%), [1]	5(1.57%), [5]	0.3710§§
Any SAE in TEAE	0.63%), [1]	0(0.00%), [0]	1(0.31%), [1]	1.0000§§
Any SADR in TEAE	0(0.00%), [0]	0(0.00%), [0]	0(0.00%), [0]	NA

Abbreviations: N=Number of Safety Set, n: Number of subjects with at least one event in the category, AE: Adverse Event; TEAE: Treatment Emergent Adverse Event; SAE: Serious Adverse Event; ADR: Adverse Drug Reaction
Note1: All AE is coded using the MedDRA version 24
Note2: If a subject has multiple occurrences of an AE after investigational product(IP) administration, the subject in presented only once in the respective subject count(n)
p-value is calculated on chi-square(§) test or Fisher's exact test(§§)

Table S5. illustrates Adverse Drug Reaction (ADRs) in TEAE.

**SOC**<br>PT	**MBA-P01**<br>**N=249**<br>**n(%)[case]**	**ONA-BoNT-A**<br>**N=159**<br>**n(%)[cases]**	**Total**<br>**N=319**<br>**n(%)[cases]**	***p*-value**
Total	4(2.52%), [4]	1(0.63%), [1]	5(1.57%), [5]	0.3710§§
**Eye disorders**	**2(1.26%), [2]**	**0(0.00%), [0]**	**2(0.63%), [2]**	
Eyelid sensory disorder	2(1.26%), [2]	0(0.00%), [0]	2(0.63%), [2]	
**General disorders and administration site conditions**	**1(0.63%), [1]**	**1(0.63%), [1]**	**2(0.63%), [2]**	
Injection site reaction	1(0.63%), [1]	1(0.63%), [1]	2(0.63%), [2]	
**Nervous system disorders**	**1(0.63%), [1]**	**0(0.00%), [0]**	**1(0.31%), [1]**	
Headache	1(0.63%), [1]	0(0.00%), [0]	1(0.31%), [1]	

Abbreviations: N=Number of subjects in a specific group, n: Number of subjects in the specific category, SOC: System Organ Class, PT: Preferred Term, [case]: Number of cases
Note1: AE is coded using MedDRA version 24.0

Table S6. illustrates adverse events and severity summary.

	**MBA-P01**<br>**N=249**<br>**n(%)[case]**	**ONA-BoNT-A**<br>**N=159**<br>**n(%)[cases]**	**Total**<br>**N=319**<br>**n(%)[cases]**	***p*-value**
Total	30(18.87%), [45]	27(16.98%), [39]	57(17.92%), [84]	0.6609§
MILD	22(13.84%), [36]	26(16.35%), [35]	48(15.09%), [71]	
MODERATE	6(3.77%), [7]	1(0.63%), [4]	7(2.20%), [11]	
SEVERE	2(1.26%), [2]	0(0.00%), [0]	2(0.63%), [2]	

Abbreviations: N=Number of Safety Set, n: Number of subjects with at least one event in the category Note1: Adverse Events are coded using the MedDRA version 24 Note2: AEs in the tables are analyzed based on TEAE tables

p-value is calculated on chi-square(§) test or Fisher's exact test(§§)
